# Circ-ASH2L promotes tumor progression by sponging miR-34a to regulate Notch1 in pancreatic ductal adenocarcinoma

**DOI:** 10.1186/s13046-019-1436-0

**Published:** 2019-11-12

**Authors:** Yan Chen, Zhonghu Li, Mengyun Zhang, Bo Wang, Jiaxin Ye, Yang Zhang, Di Tang, Dandan Ma, Weidong Jin, Xiaowu Li, Shuguang Wang

**Affiliations:** 1 Hepatobiliary Surgery Institute, Southwest Hospital, Army Medical University, Chongqing, China; 2 Hepatobiliary Surgery Department, 985 Hospital of PLA, Chongqing, Chongqing, China; 3 Dept. general surgery, Central Theater Command General Hospital of PLA, Hubei, China; 4 Department Rheumatology of Integrated Traditional Chinese and Western Medicine, Central Theater Command general hospital of PLA, Hubei, China; 5 Hepatobiliary Surgery & Carson International Cancer Shenzhen University General Hospital & Shenzhen University Clinical Medical Academy Center Shenzhen University, Shenzhen, China

**Keywords:** Circular RNA, miR-34a, Notch1, Pancreatic ductal adenocarcinoma, Tumor progression, Metastasis, Prognosis

## Abstract

**Background:**

Circular RNAs (circRNAs) have recently been shown to play important roles in different tumors. However, their detailed roles and regulatory mechanisms in pancreatic ductal adenocarcinoma (PDAC) are not well understood. This study aimed to identify enriched circRNAs and detect their functions and mechanisms in PDAC cells and tissues.

**Methods:**

circRNA-ASH2L (circ-ASH2L) was identified by circRNA microarray studies based on previous studies, and further detected in PDAC cells and samples by qRT-PCR. The functions of circ-ASH2L were identified by transwell, EdU, cell cycle or Tube formation assays. The regulatory mechanisms of circ-ASH2L were explored by WB, RIP, FISH, dual-luciferase assays, RNA pulldown or other assays.

**Results:**

We identified a circRNA (circ-ASH2L) based on our previous studies, detected its expression in different malignant cells and found that circ-ASH2L was highly expressed in pancreatic cells or tumor tissues and correlated with tumor malignancy. Further studies revealed that circ-ASH2L promoted tumor invasion, proliferation and angiogenesis by regulating miR-34a, thus regulate Notch 1 expression. Circ-ASH2L served as a miRNA sponge for miR-34a and promoted tumor progression in vivo. Finally, we analyzed circ-ASH2L expression in clinical tissues and found that high circ-ASH2L expression was correlated with lymphatic invasion and TNM stage and was an independent risk factor for pancreatic patient survival.

**Conclusions:**

circ-ASH2L play an important role in tumor invasion, and high circ-ASH2L may be a useful marker of PDAC diagnosis or progression.

## Background

Pancreatic ductal adenocarcinoma (PDAC) is undoubtedly one of the most aggressive and deadliest malignancies and the fourth leading cause of cancer-related death [1, 2]. Despite decades of effort, the outcome of PDAC remains disappointing, with the 5-year survival rate being only approximately 6% [2]. Surgical resection remains the only potentially curative therapy, but most patients are diagnosed at advanced stages and miss their chance for an operation due to a lack of specific symptoms and effective tumor-related markers [3, 4]. The lack of markers for detecting patients with a high risk of tumor metastasis and recurrence also leads to the disappointing prognosis of PDAC patients. Thus, it is of paramount importance to identify effective and stable diagnostic and prognostic markers for clinical management of PDAC.

Circular RNAs (circRNAs), which are mainly distributed in the cytoplasm, represent a novel class of widespread and abundant transcripts that form a covalently closed continuous loop [5]. Initially, circRNAs were regarded as the by-products of splicing or splicing errors with low abundance [6]; However, more and more circRNAs have been successfully identified in multiple cell lines or across various species recently [7–9]. CircRNAs can regulate gene expression through interactions with microRNAs (miRNAs) by acting as miRNA sponges or translating proteins directly in various biological activities [10, 11]. In recent years, increasing evidence has suggested that aberrant circRNA expression may contribute to the development of different cancers, such as lung cancer [12, 13], esophageal squamous cell carcinoma [14], hepatocellular cancer [15], colorectal cancer and so on [16, 17]. These observations indicate that circRNAs may be a new class of potential biomarkers or therapeutic targets for cancer [7]. However, pancreatic cancer-related circRNAs have not been fully elucidated and should be further studied.

MicroRNAs (miRNAs) are 21–25 nucleotide noncoding RNAs that can post-transcriptionally downregulate the expression of various target genes [18]. Deregulation of miRNAs, was shown to play pivotal roles in tumorigenesis and progression. Different types of miRNAs were identified in the past decades, and lots of miRNAs were found to be involved in nearly the whole biological processes of tumor initiation and progression, such as MiR-34a was reported to play important roles in multiple types of cancer including prostate cancer, neuroblastoma tumor, hepatocellular carcinoma and so on [19]. By binding to the 3’UTR of target genes, miRNAs induce mRNA degradation and/or translational inhibition [20]. Thus, miRNAs could act as tumor promoter or suppressor depend on their regulated gene or genes. In pancreatic cancer, tumor prognosis related miRNAs are still lacking and need to be further detected.

In this study, we identified a new circ-RNA, circ-ASH2L, in PDAC; its cellular roles and regulatory mechanisms were also determined, and we further tested the roles of circ-ASH2L in animal experiments. Finally, we analyzed circ-ASH2L expression in clinical tumor samples and showed that circ-ASH2L may have important functions in PDAC.

## Materials and methods

### Cell culture and transfection

The Pancreatic ductal adenocarcinoma (PDAC) cell lines AsPC-1, BxPC-3, Capan-1, Hs 766 T, PANC-1 and SW1990 were purchased from ATCC, HEK 293 and normal pancreatic cell HPDE were also purchased from ATCC. Hs 766 T-L1, Hs 766 T-L2 and Hs 766 T-L3 are the first, second and third generation primary cells from liver metastatic tissue of Hs 766 T as described in our previous papers [21]. PDAC cells were cultured in RPMI-1640 medium (Gibco, USA) and HEK-293, HPDE were cultured in DMEM supplemented with 10% fetal bovine serum (FBS) (Gibco, USA) at 37 °C in a humidified atmosphere containing 5% CO_2_. Cell transfections were carried out as described previously [21]. Briefly, for lentivirus transduction, 10^5^ PDAC cells were incubated in a 6-well plate with 2 ml of medium containing 100 μl (10^7^ U) of lentivirus particles and 5 μg/ml polybrene for 24 h. Plasmid, miR-34a and shRNA transfections were performed using Lipofectamine 3000 (Invitrogen, USA) according to the manufacturer’s instructions.

### PDAC patients and clinical samples

A total of 90 patients (please refer to our previous study [9]) underwent pancreaticoduodenectomy surgery at Department of Hepatobiliary Surgery Institute, Southwest Hospital, from January 2012 to January 2016. All patients were confirmed to have PDAC by ultrasonography, contrast-enhanced CT or MRI examination and a blood test for markers of digestive system tumors. For the clinical characteristics of these patients, please refer to Table 1. A total of 90 fresh frozen tissues and 25 peritumoral normal tissues were used for RNA isolation, fresh tissues were put into liquid-nitrogen immediately after tumor excision and then transferred to − 80 °C for future use. Especially, each extracted RNA sample was subjected to an agarose electrophoresis, any sample whose 28 s rRNA bind vanished was excluded from this study in case of RNA degradation. All patients were followed up by radiography, ultrasonography or CT examination every 3 months after discharge and were followed up monthly by telephone in the clinical follow up center of the Department of Hepatobiliary Surgery Institute, Southwest Hospital. This study was approved by the Ethics Committee of Southwest Hospital, and all patients provided written informed consent, and the study protocol conformed to the ethical guidelines of the Declaration of Helsinki (1975).

**Table 1:** Clinical characteristics and expressions of circ-ASH2L in 90 pancreatic carcinoma patients

All cases	circ-ASH2L			
	High	Low	χ^2^	*P*-value
	45	45		
Gender			0.257	0.800
Male	34	36		
Female	11	9		
Age, years			0.045	1.000
≤ 60	24	25		
> 60	21	20		
Tumor location			0.278	0.793
Head	35	37		
Body or tail	10	8		
Tumor size, cm			0.200	0.823
≤ 2	29	31		
> 2	16	14		
Neural invasion			0.000	1.000
Yes	18	18		
No	27	27		
Duodenal invasion			0.385	0.758
Yes	7	5		
No	38	40		
Differentiation			3.090	0.213
Low	12	8		
Median	30	29		
High	3	8		
Lymphatic invasion			5.954	**0.015**
Yes	21	10		
No	24	35		
Vascular invasion			2.862	0.091
Yes	15	8		
No	30	37		
Liver metastasis			3.462	0.063
Yes	9	3		
No	36	42		
T factor			3.629	0.057
T1,2	20	29		
T3,4	25	16		
TNM			7.647	**0.006**
I or IIA	19	32		
IIB or III, IV	26	13		

#The median expression level of each circ-RNA was used as the cut-off value. The correlation analysis was tested by chi-square tests. The bold values indicate *P*-values less than 0.05

### RNase R treatment

The treatment of RNase R was carried out according to the manufacturer’s instructions. Briefly, total RNA (2 μg) after be extracted by Trizol reagent was incubated with 3 U/μg of RNase R (Epicentre Technologies, USA) for 20 min at 37 °C, then treated RNA was purified with an RNA clean kit (DP412, TIANGEN, China) according to the instructions.

### Cell migration and invasion assay

Cell migration was examined by a wound-healing assay according to previously described [21]. Briefly, cells were cultured regularly until reaching confluence and the medium was replaced with serum-free 1640 medium, wounds were made with a 10 μl-pipette tip and photos were captured by phase-contrast microscope. For cell invasion assay, 5 × 10^5^ PDAC cells in 300 μl of serum-free medium were cultured in a chamber containing an 8 μm polycarbonate filter (Millipore, USA) coated with 30 μl of Matrigel (BD, USA), cells remaining on the upper membrane were stained with 0.5% crystal violet at last. All the statistical results were obtained from three independent experiments averaged from five randomly selected image fields.

### Tube formation assay

The formation of capillary-like structures was assessed in a 24-well plate using growth factor–reduced Matrigel (BD, CA, USA). 4 × 10^4^ cells HUVECs were plated on top of Matrigel (280 ml/well) after transfection. After 24 h, cells were visualized under the contrast phase microscope. The total tube area was quantified as mean pixel density obtained from image analysis of three random microscopic fields using ImageJ software.

### 5-Ethynyl-2′-deoxyuridine (EdU) incorporation assay

The EdU assay was performed with a keyFluor488 Click-iT EdU detection kit (KGA331–100, KeyGene, Nanjing, China) according to the manufacturer’s instructions as previously described [22]. Briefly, PADC cells were seeded on cell slides, after the treatment of circ-RNA or miR-34a transfection, cells were incubated with 50 mM EdU for 2 h, then cells were fixed and permeabilized, then cells were incubated with Click-It reaction mixture, the slides were mounted with Mounting Medium for Fluorescence with DAPI and imaged with a fluorescence microscope.

### Western blot analysis

Procedures of WB assays were conducted as previously described [9]. Briefly, total protein of PDAC cells was extracted by RIPA lysis buffer (Thermo, USA). After measured by BCA Protein Assay Kit (Beyotime, China), 30 μg protein were used to SDS-page and transferred to PVDF membranes (Millipore, USA), which were blocked and blotted with primary antibodies overnight at 4 °C. The antibodies used in this study included the following: anti-Notch1 (1:1000, #3608, CST, USA) and anti-β-actin (1:5000, 20,536–1-AP, Proteintech, USA). The membranes were washed with PBST and incubated with horseradish peroxidase-conjugated secondary antibody for 2 h and the immunocomplexes were then visualized using a New Super ECL Detection Kit (KeyGEN BioTECH, China) according to the manufacturer’s protocol.

### RNA isolation and qRT-PCR analysis

Cellular RNA was isolated using TRIzol reagent (Thermo, USA). First-strand cDNA was generated with PrimeScript RT Reagent Kit with gDNA Eraser (TaKaRa, Japan) and miRNA reverse transcription was performed using a Mir-X miRNA qRT-PCR SYBR Kit (Clontech, Japan). Real-time PCR was performed using the PrimeScript RT Reagent Kit and SYBR Premix Ex Taq (TaKaRa, Japan) on a CFX96 Real-Time System (Bio-Rad, USA) with the reaction conditions provided in the instructions. The primer details used in the study were shown below:

**Table:** 

RNAs	Sequence	Sequence	
circ-ASH2L	F: AACCAAGTTCCACCAGTCCA	R: CGGTATCTGGTGGCATCTCA	
β-actin	F: GCGGACTATGACTTAGTTGCGTTACA	R: TGCTGTCACCTTCACCGTTCCA	
P21	F: GTCCAGCGACCTTCCTCATCCA	R: CCATAGCCTCTACTGCCACCATCT	
c-Met	F: GTCGCTCCGTATCCTTCTCTGTTG	R: GCCTCTGGTTCTGATGCTCTGTC	
SNAIL	F: GGCTCCTTCGTCCTTCTCCTCTAC	R: GTGGCTTCGGATGTGCATCTTGA	
Notch1	F: TGCGAGACCAACATCAACGAGTG	R: TCAGGCAGAAGCAGAGGTAGGC	
U6	F: CTCGCTTCGGCAGCACA	R: AACGCTTCACGAATTTGCGT	
miR-34a	GGTGGCAGTGTCTTAGCTGG	miR-128a	CCCTCACAGTGAACCGGTC
miR-605-5p	GGTAAATCCCATGGTGCCTTC	miR-885-3p	AGGCAGCGGGGTGTAGT
miR-142-3p	GGGTGTAGTGTTTCCTACTTTATGG		

Additionally, the miRNA mRQ 3′ primer was provided in the Mir-X miRNA qRT-PCR SYBR Kit (Clontech, Japan).

### Flow cytometric analysis

The procedures of cell cycle analysis were referred to the manufacturer’s instructions of Cell Cycle Detection Kit (KGA512, KeyGEN BioTECH, China). Basically, PDAC cells were fixed in 70% ethanol at 4 °C overnight. Then, cells were added to RNase A and propidium iodide (PI) at dark for 30 min. Cell cycle distribution was assessed by a FACScan flow cytometer (BD Biosciences, NJ, USA). The percentage of cells was investigated by FlowJo (USA).

### Dual-luciferase reporter assay

5 × 10^3^ cells were cultured in a white 96-well plate and then transfected with psiCHECK2-circ-ASH2L or psiCHECK2-circ-ASH2L mut plasmid (Sangon Biotech, China) and 8 ng of the internal control pRL-TK Renilla luciferase plasmid (Promega, USA), together with miR-34a (RiboBio, China) oat a final concentration of 0, 50 or 150 nM. After a 48-h incubation, the cells were harvested and processed with the Dual-Luciferase Reporter Assay System (E1910, Promega, USA) according to the manufacturer’s protocol. The results were quantified as the ratio of firefly luciferase activity/Renilla luciferase activity in each well.

### Animal experiment

All animal experiments were approved by the Institutional Animal Care and Use Committee of Southwest Hospital, Chongqing, China. Briefly, Four- to six-week-old male randomly selected athymic nude mice were obtained from Southwest Hospital (Chongqing, China) and housed in the standard pathogen-free conditions of Southwest Hospital (Chongqing, China). The mice were anesthetized by an intraperitoneal injection of 1% pentobarbital sodium (50 mg/kg). A median abdominal incision was made to expose the spleen and pancreas, and 5 × 10^6^ PDAC cells (in 100 μl of PBS) were injected to the head of the pancreas. After replacing the pancreas and closing the abdomen, the mice were imaged by the IVIS Lumina II system (Caliper Life Science, USA).

### RNA-binding protein immunoprecipitation (RIP) assay

The immunoprecipitation of the circ-ASH2L bound to Ago2 was performed using a Magna RIP™ RNA-Binding Protein Immunoprecipitation Kit (Merck Millipore, Germany). 2 × 10^7^ HEK-293 cells were harvested in RIP lysis buffer and the lysates were stored at − 80 °C. 8 μg of anti-Ago2 (MA5–23515, Invitrogen, USA) or normal control IgG was incubated with magnetic beads for 2 h at RT and then 100 μl of the supernatant of RIP lysate was mixed with 900 μl of RIP immunoprecipitation buffer and added to the bead-antibody complexes to incubate overnight at 4 °C. The beads were further mixed with proteinase K buffer and incubated for 30 min at 55 °C and RNA was finally extracted for PCR use.

### Biotinylated RNA pulldown assays

For biotin-coupled miR-34a pull-down assay, the procedures were similar with our previous study [9]. HEK 293 cells were seeded and cultured in 10-cm dish, then 50 μM biotinylated miR-34a or biotinylated miR-Scr (as NC) were transfected with standard protocol of lipo3000 (L3000015, Invitrogen, thermo, USA), then cells were harvested and washed in cold PBS, lysed in lysis buffer. Streptavidin-conjugated magnetic beads were activated and blocked with blocking buffer for 2 h, then beads were added to cell lysates for incubation overnight at 4 °C. Then beads were washed with lysis buffer and its RNAs were extracted by TRIzol LS (10,296,028, Invitrogen, Thermo, USA). Then RNAs were reverse transcripted by RT reagent Kit (RR047A, Takara, Japan) and analyzed by qRT-PCR assay.

### Fluorescence in situ hybridization

Capan-1 cells were cultured on cell slides and then treated with plasmids or other methods, next cells were fixed with 4% paraformaldehyde, permeabilized with 0.5% Triton X-100 (Biosharp, China) and then processed using a Ribo™ Fluorescent In Situ Hybridization Kit (RiboBio, China). To be short, cells were first blocked with pre-hybridization buffer for 30 min at 37 °C and incubated with a circ-RNA or 18 s control FISH Probe Mix and hybridization buffer overnight at 37 °C, then slides were washed with hybridization washing buffer I, II and III for 15 min at 42 °C and 0.2 × SSC at 53 °C.

### Statistical analysis

The correlation between clinical categorical parameters and circ-ASH2L expression (the median was regard as cutoff value) was evaluated by a χ^2^ test. Student’s t-test was used to compare group differences if they followed a normal distribution. One-way ANOVA was applied to compare the differences among 3 groups. For survival analysis, univariate analysis was conducted by the KM method (the log-rank test), and multivariate analysis was performed by the stepwise Cox multivariate proportional hazard regression model (Forward LR, likelihood ratio). All analyses were performed using SPSS 22.0 software (IBM, USA), all the tests are two-sided and a *P*-value < 0.05 was considered to be statistically significant. All statistical analyses were completed under the guidance of experienced experts from the Statistics Department of the Army Medical University.

Additionally, all methods were carried out in accordance with relevant guidelines and regulations!

## Results

### The identification of circ-ASH2L

**Fig. 1:**
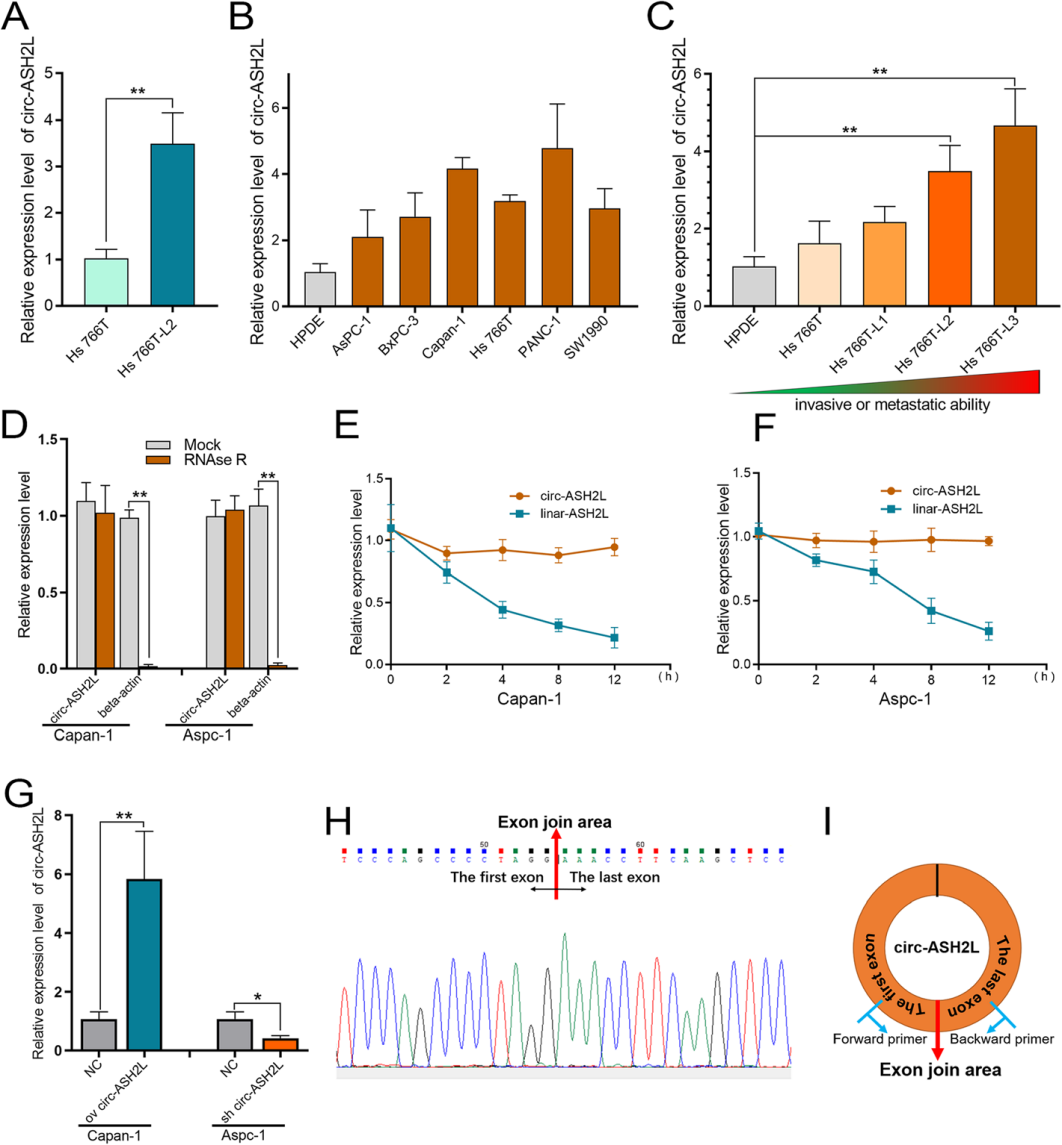
**a** Relative circ-ASH2L expressions of Hs 766 T and Hs 766 T-L2 were measured by qRT-PCR. **b** Relative circ-ASH2L expressions of HPDE (the normal pancreatic cell line) and the indicated PDAC cells were measured by qRT-PCR. **c** Relative circ-ASH2L expressions of HPDE (the normal pancreatic cell line) and the indicated PDAC cells were measured by qRT-PCR. **d** qRT-PCR analysis of circ-ASH2L or β-actin after treatment of RNase R in Capan-1 and Aspc-1 cells. **e** qRT-PCR analysis of circ-ASH2L and β-actin after treatment of Actinomycin D at the indicated time points in Capan-1 cells. **f** qRT-PCR analysis of circ-ASH2L and β-actin after treatment of Actinomycin D at the indicated time points in Aspc-1 cells. **g** Relative expressions of circ-ASH2L in indicate treated Capan-1 and Aspc-1 cells was measured by qRT-PCR. **h** The PCR products of Capan-1 cells were confirmed by Sanger sequencing to show the back-splice junction. **i** Schematic outlining the details of circ-ASH2L and its primer-designing details

In our previous studies, we identified several circ-RNAs that were closely associated with tumor progression and prognosis in tumor exosomes [9]. However, we also found that a circ-RNA, circ-ASH2L, was abundant only in tumor cells, rather than in tumor exosomes. We investigated whether circ-ASH2L plays an important role in PDAC progression. First, we verified circ-ASH2L expression in PDAC cells. The qRT-PCR results indicated that circ-ASH2L showed low expression, which was similar in the exosomes of Hs 766 T and Hs 766 T-L3 cells (data not shown), but circ-ASH2L expression in Hs 766 T-L2 cells was approximately 3.3-fold higher than that in Hs 766 T cells (Fig. 1a). We further examined the results in HPDE (a normal pancreatic epithelial cell line) and six PDAC cell lines, and the results indicated that circ-ASH2L was barely expressed in HPDE cells, but its expression was much higher in PDAC cells (Fig. 1b). To further probe whether circ-ASH2L was related to tumor malignancy, circ-ASH2L expression was detected in Hs 766 T (the parent cell), Hs 766 T-L1, Hs 766 T-L2 and Hs 766 T-L3 (the daughter cell) cells (Hs 766 T-L1, Hs 766 T-L2 and Hs 766 T-L3 cells are the first, second and third-generation primary tumor cells, respectively, with increasing invasive ability and were derived from a liver metastasis of Hs 766 T cells); as the malignancy of PDAC cells increased, the expression levels of circ-ASH2L increased successively (Fig. 1c). We then analyzed the circ-ASH2L in Capan-1 and Aspc-1 cells. We found that circ-ASH2L, but not β-actin, could resist digestion by RNase R (Fig. 1d). When PDAC cells was treated with Actinomycin D, a transcription inhibitor, we found that circ-ASH2L was much more stable than linar-ASH2L (Fig. 1e-f). We next constructed the overexpression plasmid or shRNA of circ-ASH2L and confirmed the upregulation or downregulation of this molecule by qRT-PCR in Capan-1 and Aspc-1 cells (Fig. 1g). To confirm the characteristics of circ-ASH2L, the PCR products of PDAC cells were confirmed by Sanger sequencing to show the back-splice junction (Fig. 1h), and the details of circ-ASH2L and its primer-designing details were outlined in Fig. 1i. Thus, we showed that circ-ASH2L was abundant, stable and highly expressed in PDAC cells.

### Circ-ASH2L promotes tumor invasion, proliferation and angiogenesis in PDAC

**Fig. 2:**
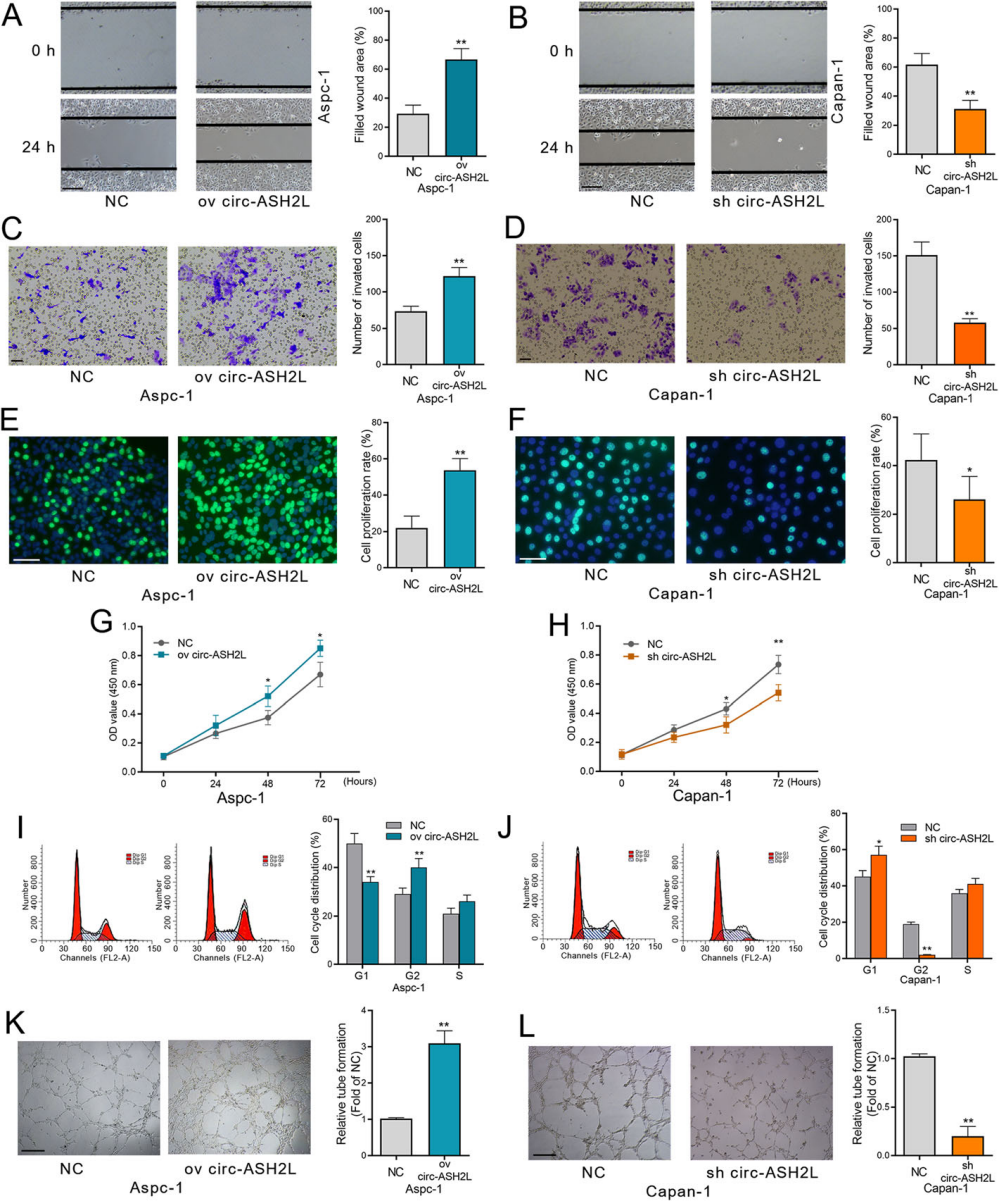
**a-b** The migration abilities of indicate treated Aspc-1 (**a**) and Capan-1 cells (**b**) were measured by wound-healing assays. Scale bars = 50 μm. **c-d** The invasion abilities of indicate treated Aspc-1 (**c**) and Capan-1 cells (**d**) were measured by transwell assays. Scale bars = 50 μm. **e-f** The proliferation abilities of indicate treated Aspc-1 (**e**) and Capan-1 cells (**f**) were measured by EdU assays. Scale bars = 50 μm. **g-h** The proliferation abilities of indicate treated Aspc-1 (**g**) and Capan-1 cells (**h**) were measured by CCK-8 assays daily for 3 days. **i**-**j** The indicate treated Aspc-1 (**i**) and Capan-1cells (**j**) were stained by propidium iodide and analyzed using flow cytometry. **k-l.** The in vitro angiogenesis abilities of indicated treated Aspc-1 (**k**) and Capan-1 cells (**l**) were measured by tube-formation assays of HUVECS cells. Scale bars = 50 μm

The results above suggest that circ-ASH2L was correlated with tumor malignancy; thus, we investigated the specific cellular functions of circ-ASH2L in PDAC cells. First, wound healing and transwell assays showed that upregulating circ-ASH2L promoted the migration or invasion of Capan-1 and Aspc-1 cells, and these functions could be blocked by circ-ASH2L inhibition (Fig. 2a-d). Similarly, circ-ASH2L could also promote cell proliferation, as shown by EdU and CCK-8 assays of Capan-1 and Aspc-1 cells (Fig. 2e-h). Moreover, Flow cytometry analysis showed that less cells stopped in G1 phase after overexpressing circ-ASH2L in Aspc-1 cells (Fig. 2i), but silencing of circ-ASH2L arrested cell cycle in G1 phase in Capan-1 cells (Fig. 2j). We further tested the effects of circ-ASH2L in angiogenesis. When HUVECs were transfected with circ-ASH2L, the tube-like structures were significantly enhanced compared to the normal or sh-circ-ASH2L groups (Fig. 2k-l). Together, the results above suggest that circ-ASH2L could promote invasion, proliferation and angiogenesis in PDAC.

### Circ-ASH2L acts as a sponge for miR-34a

**Fig. 3:**
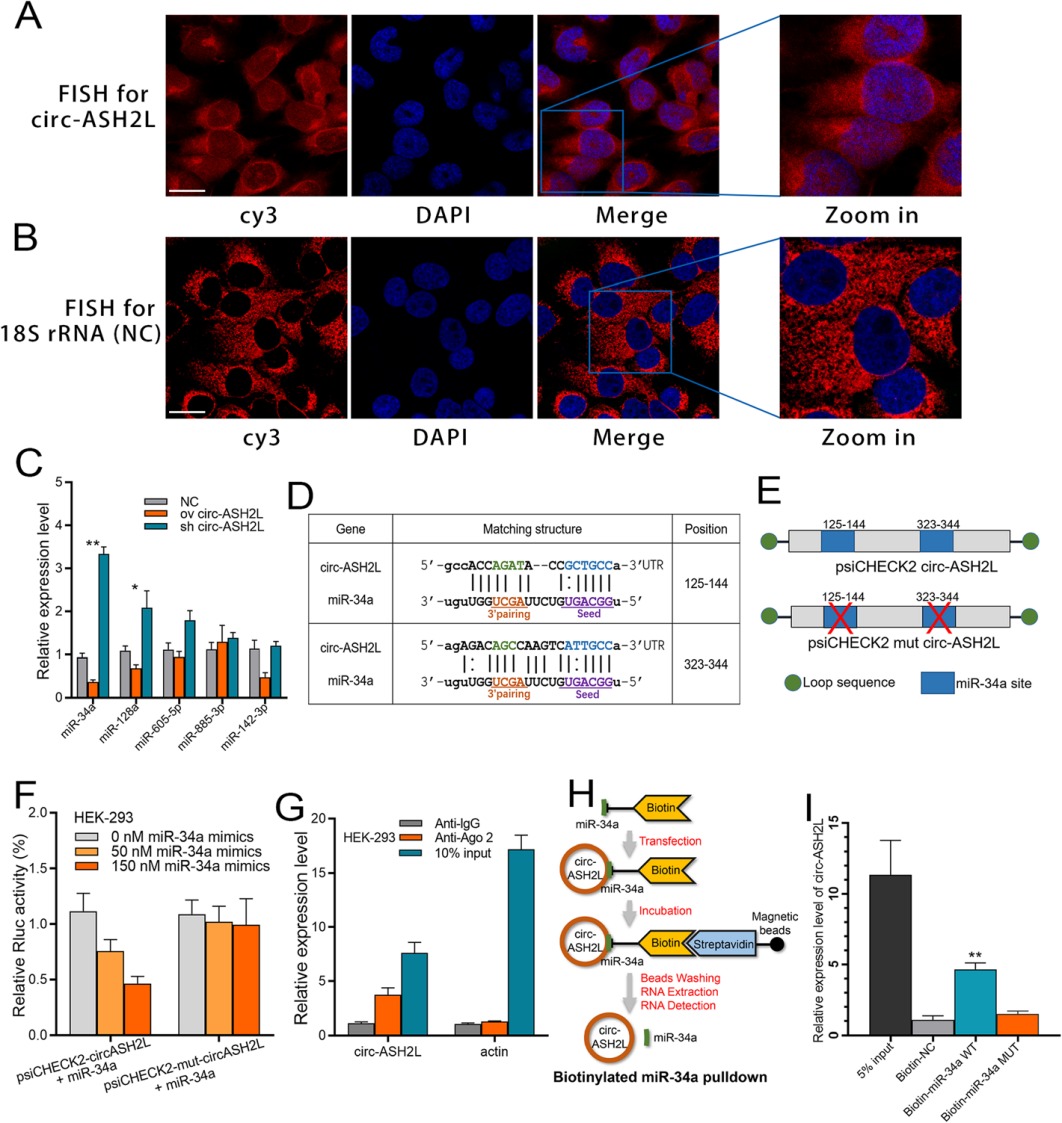
**a-b** The in-situ expressions of circ-ASH2L (**a**) and 18S (**b**, as a control) in Capan-1 cells. Scale bars = 12.5 μm. **c** The expressions of indicated miRNAs in indicate treated HEK-293 cells were measured by qRT-PCR. **d** The prediction for miR-34a binding sites on circ-ASH2L transcript. **e** Schematic outlining the wild type and mut circ-ASH2L luciferase plasmid. (**f**) Luciferase activity in HEK-293 cells co-transfected with indicated concentration of miR-34a or circ-ASH2L luciferase reporter transcript. Data are showed as the ratio of firefly activity to Renilla luciferase activity. **g** RNA immunoprecipitation (RIP) experiments were performed using the anti-Ago2 or lgG antibody to immunoprecipitates, the expressions of circ-ASH2L or β-actin were measured by qRT-PCR. **h** The schematic diagram of RNA pull-down assay in this study. **i** circ-ASH2L was pulled down and enriched with miR-34a probe and then detected by qRT-PCR

Although some circ-RNAs can be translated into proteins, the main regulatory mechanism of circ-RNA is to function as a miRNA sponge in the cytoplasm. Therefore, we first investigated whether circ-ASH2L transcripts were located in the cytoplasm. Fluorescence in situ hybridization (FISH) results showed that the fluorescence signals of the circ-ASH2L transcript were similar to those of the control, 18S rRNA: most of the circ-ASH2L transcript signals were located in the cytoplasm of Capan-1 cells, with very few hybridization signals observed in the nuclear region (Fig. 3a-b). Furthermore, we predicted the potential circ-ASH2L sponge miRNAs using the miRanda tool, and the top 5 possible miRNAs were assessed by qRT-PCR analysis. The results showed that upregulating circ-ASH2L decreased the expression of these miRNAs, while downregulating circ-ASH2L increased the expression of these miRNAs. Although the candidate miRNAs showed similar effects, miR-34a was the most significant (Fig. 3c); thus, we hypothesized that miR-34a is the most likely miRNA sponged by circ-ASH2L. A detailed bioinformatics analysis revealed that each circ-ASH2L transcript has two strong miRNA binding sites, which were all located in the seed zone (Fig. 3d). To further confirm this speculation, wild or mutated (sequences of the sponge sites were deleted) dual luciferase plasmids were constructed (Fig. 3e) and co-transfected with miR-34a mimics into HEK-293 cells. The dual-luciferase reporter assay showed that the wild type plasmid group showed decreased Rluc expression, while the mutated group did not. These results suggest that circ-ASH2L may sponge miR-34a transcripts and thus decrease Rluc fluorescence signals (Fig. 3f). The next RNA immunoprecipitation (RIP) assay showed that circ-ASH2L was enriched in Ago2-containing immunoprecipitates compared to actin (Fig. 3g). Biotin-coupled miR-34a pulldown assay showed that circ-ASH2L was detected in the miR-34a WT pulled down pellet compared with the NC or miR-34a MUT group (Fig. 3h-i). The results above indicate that circ-ASH2L could act as a sponge for miR-34a.

### Circ-ASH2L promotes tumor progression via miR-34a

**Fig. 4:**
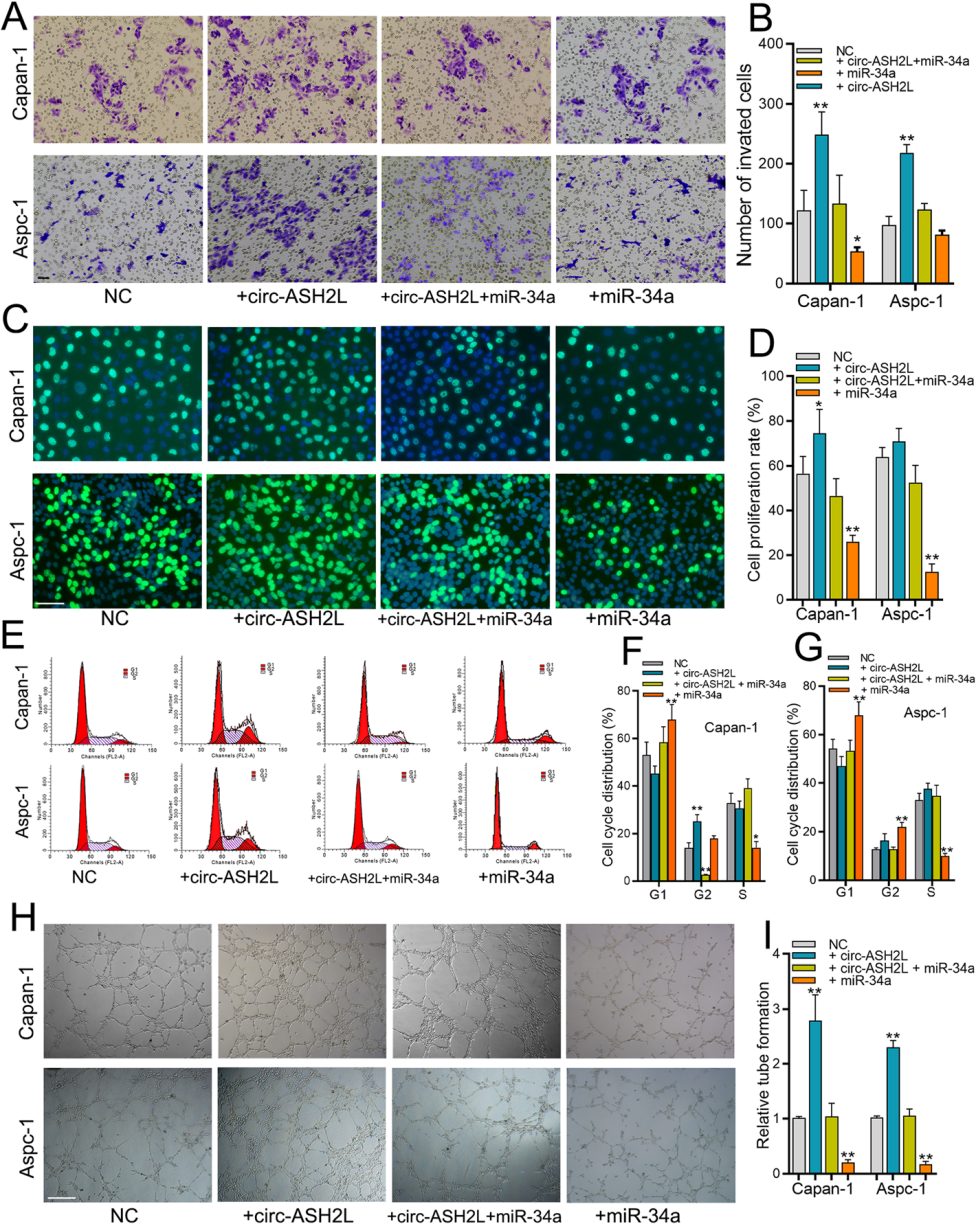
**a-b** The invasion abilities of indicate treated Capan-1 (**a**) and Aspc-1 cells (**b**) were measured by transwell assays. Scale bars = 50 μm. **c-d** The proliferation abilities of indicate treated Capan-1 (**c**) and Aspc-1 cells (**d**) were measured by EdU assays. Scale bars = 50 μm. **e-g** The indicate treated Aspc-1 and Capan-1 cells (**e**) were stained by propidium iodide and analyzed using flow cytometry, the statistical results of Aspc-1 (**f**) and Capan-1 (**g**) cells were showed in right column. **h-i** The in vitro angiogenesis abilities of indicated treated Capan-1 and Aspc-1 cells were measured by tube-formation assays of HUVECS cells (**h**), and the statistical result was showed in right column (**i**)

The main functions of miR-34a are regulating the cell cycle, cellular proliferation, apoptosis and angiogenesis in tumors as reported by previous studies [19, 23]. As the cellular functions of circ-ASH2L and miR-34a are quite similar, and we demonstrated that circ-ASH2L acts as a sponge for miR-34a, we investigated whether circ-ASH2L promotes tumor progression via miR-34a. Transwell and EdU assays showed that circ-ASH2L promoted tumor invasion and proliferation, while these functions could be blocked by adding a miR-34a inhibitor (Fig. 4a-d). Flow cytometry analysis showed that circ-ASH2L decreased G1 phase after overexpressing circ-ASH2L, and the decrease was blocked by miR-34a addition in Capan-1 and Aspc-1 cells (Fig. 2e-g). Similarly, circ-ASH2L overexpression cells promoted in vitro tube formation, but this promotion was decreased by transfection of miR-34a mimics (Fig. 4h-i). Together, the results above suggest that circ-ASH2L promotes tumor progression via miR-34a.

### Circ-ASH2L regulate notch 1 expression via miR-34a

**Fig. 5:**
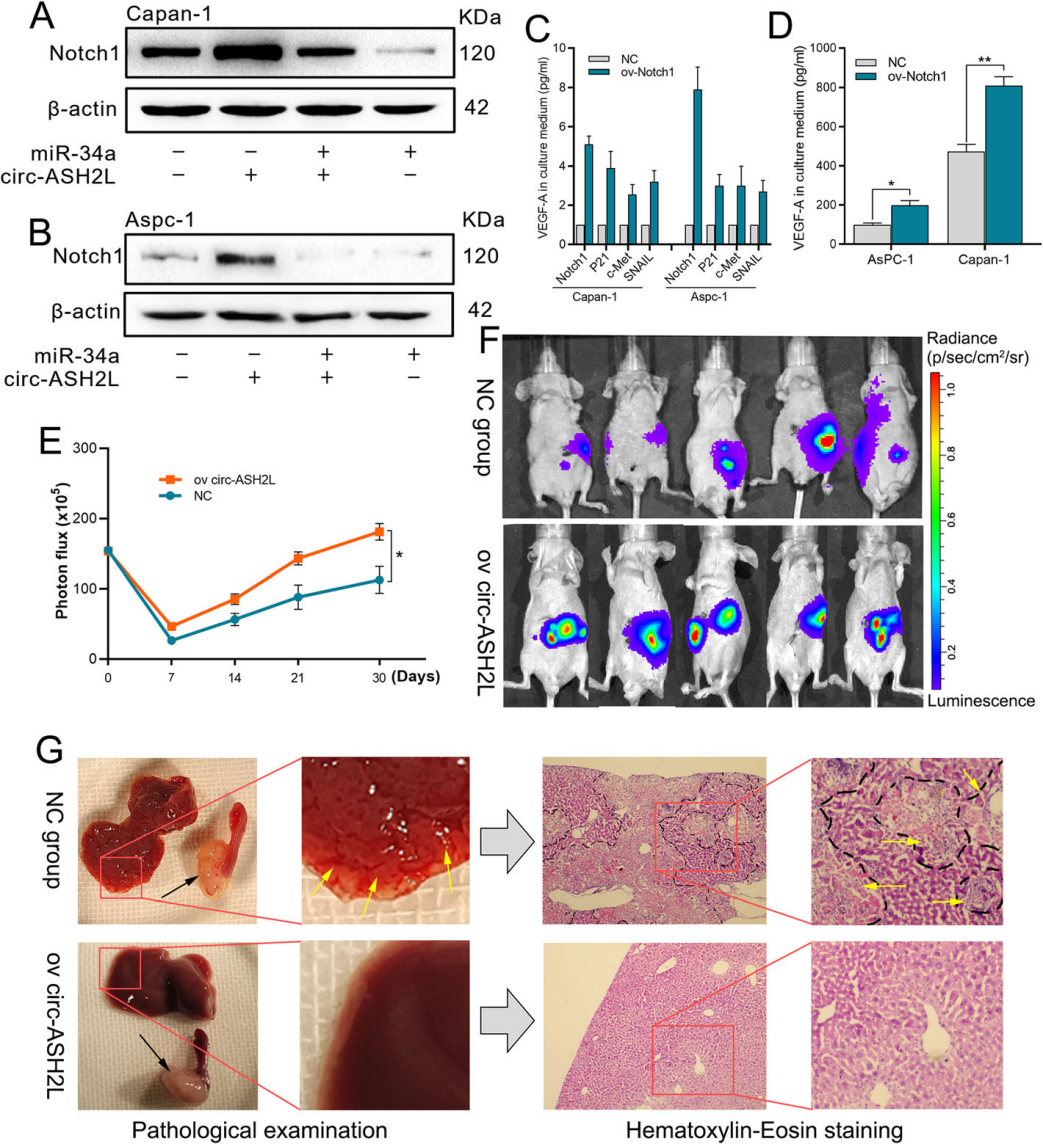
**a-b** The protein expression levels of indicate treated Capan-1 (**a**) and Aspc-1 (**b**) cells were measured by WB analysis. **c** The mRNA expression levels of indicate treated Capan-1and Aspc-1 cells were measured by qRT-PCR analysis. **d** The protein expression levels of indicate treated Capan-1 and Aspc-1 cells were measured by ELISA analysis. **e-g** Animal experiments, the luciferase intensities were measured each week (**e** and **f**) after intrapancreatic injection with NC or circ-ASH2L overexpressing Capan-1 cells, pancreatic tumor in situ (the black arrows point to) and liver metastasis foci (the yellow arrows point to) were showed by autopsy and H&E staining (**g**)

Previous studies showed that miR-34a could regulate Notch1 expression in multiple tumors, and Notch 1 signaling pathway could promote proliferation, invasion, migration, or angiogenesis in many tumors include PDAC [24–26], these functions are similar with the roles of circ-ASH2L in PDAC as mentioned above, so we suggest that circ-ASH2L may regulate Notch 1 expression via miR-34a. WB assay showed that circ-ASH2L increased Notch 1 expression but miR-34a decreased Notch 1 expression, and the miR-34a mediated inhibition can be restored by circ-ASH2L in both Capan-1 and Aspc-1 cells (Fig. 5a-b). Previous studies reported that Notch 1 can promote P21, c-Met, SNAIL or VEGF expression [23, 27, 28], Therefore, we further confirmed that in PDAC cells. qRT-PCR (Fig. 5c) or ELISA (Fig. 5d) results showed that when PDAC cells were overexpressed circ-ASH2L, P21, c-Met, SNAIL or VEGF were increased.

### Circ-ASH2L promotes tumor progression in vivo

The results above suggest that circ-ASH2L plays important roles in tumor progression in vitro. To further detect the roles of circ-ASH2 L in vivo, we established an in situ PDAC model in nude mice. First, we overexpressed circ-ASH2L in Capan-1 (ov-Capan-1) cells by circ-ASH2L lentivirus, and these cells were labeled with firefly luciferase before the circ-ASH2L transfection. Then, 10^6^ ov-Capan-1 cells were injected into the head of the pancreas of four- to six-week-old male nude mice. The luciferase intensity was assessed each week after injection to confirm tumorigenesis in situ (Fig. 5e-f). The results showed that circ-ASH2L overexpression increased tumorigenesis, as we detected intense luciferase signals at an earlier time in the ov-Capan-1 mice compared to the NC mice. Furthermore, we identified many more liver metastatic lesions in ov-Capan-1 mice than NC mice at sacrifice a month later, the liver metastatic lesions were further confirmed by H&E staining (Fig. 5g). The results above suggest that circ-ASH2L could promote tumor progression in vivo.

### High circ-ASH2L expression is an independent risk factor for PDAC survival

**Fig. 6:**
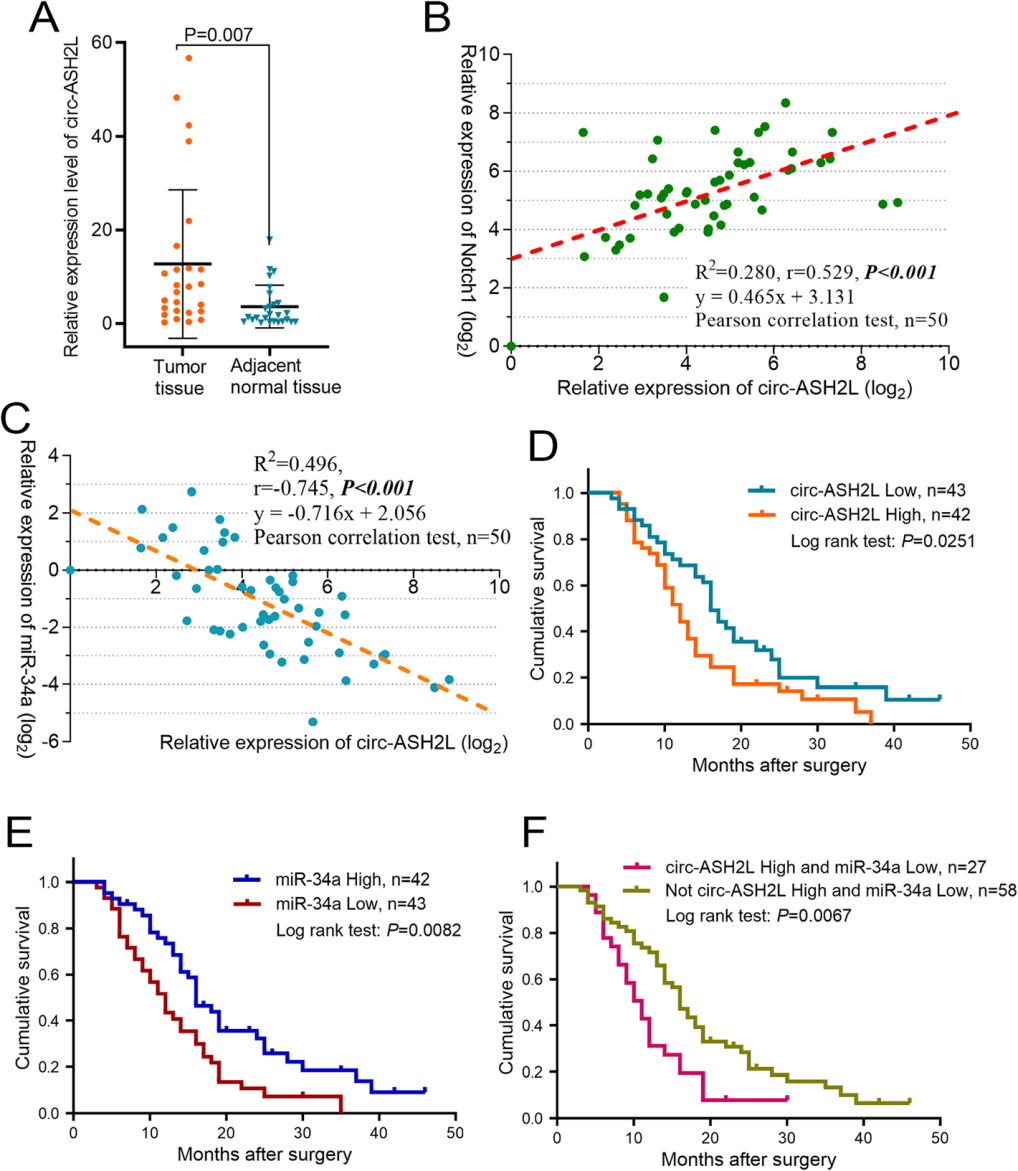
**a** The relative expression levels of circ-ASH2Lwere analyzed in 25 pairs of PDAC tumor tissues and adjacent normal tissues. **b** The correlation analysis of circ-ASH2L and Notch1 in 50 tumor tissues of PDAC patients. **c** The correlation analysis of circ-ASH2L and miR-34a in 50 tumor tissues of PDAC patients. **d-f** K-M survival curves for the overall survival of 85 PDAC patients according to the relative expression of circ-ASH2L (**d**), miR-34a (**e**) or indicate combined circ-ASH2L and miR-34a (**f**), the median expression level of each gene was used as the cut-off value to define the High or Low expression

To further detect the roles of circ-ASH2L in clinical practice. We first detected circ-ASH2L in 25 pairs of PDAC tumor tissues and their adjacent normal tissues, qRT-PCR analysis indicated that circ-ASH2L transcripts were highly expressed in PDAC tumors compared with adjacent normal tissues (Fig. 6a). To confirm the relationship of circ-ASH2L and Notch1 in vivo conditions, we detected the expressions of circ-ASH2L and Notch1 in 50 clinical PDAC tissues, the result revealed that circ-ASH2L and Notch1 were positively associated (Fig. 6b, *r* =0.529, *P* < 0.001); furthermore, we also detected the expressions of circ-ASH2L and miR-34a in 50 clinical PDAC tissues, interestingly, we found that the expression levels of circ-ASH2L and miR-34a were significantly inversely correlated (Fig. 6c, *r* = − 0.745, *P* < 0.001). We next examined the expression levels of circ-ASH2L in 90 clinical pancreatic tumor samples after pancreaticoduodenectomy and analyzed the correlations of circ-ASH2L expression to clinical parameters. We found that high circ-ASH2L expression was significantly associated with lymphatic invasion and TNM stage. However, we did not find any associations between circ-ASH2L expression and tumor size, differentiation or other parameters (Table 1). The results indicate that circ-ASH2L may also be related to poor prognosis. Thus, we further analyzed whether the expression level of circ-ASH2L was associated with the survival rate of patients with pancreatic carcinoma. KM curve analysis showed that patients with high circ-ASH2L expression had low overall survival rates (Fig. 6d). The univariate analysis results indicated that high circ-ASH2L expression, TNM stage, Vascular invasion and duodenal invasion are risk factors that affect PDAC patient survival, and multivariate analyses showed that high circ-ASH2L expression is an independent risk factors affecting PDAC patient overall survival (Table 2).

**Table 2:** Univariate and multivariate survival analyses of the prognostic factors associated with survival in pancreatic carcinoma patients (*n* = 85)

OS	Univariate analysis			Multivariate analyses		
	Patients, n	Median survial time	*P*-value	HR	95% CI	*P*-value
Gender			0.153			
Male/Female	67/18	15/12				
Age			0.4			
≤ 60/> 60	38/47	14/13				
Tumor location			0.771			
head/body or tail	67/18	14/12				
Tumor size			0.165			
≤ 2/> 2	57/28	13/16				
Neural invasion			0.345			
Yes/No	33/52	14/14				
Duodenal invasion			**0.005**	2.535	1.316–4.884	0.005
Yes/No	12/73	10/16				
Differentiation			0.585			
Low/Median/High	19/57/9	14/14/18				
Lymphatic invasion			0.184			
Yes/No	31/54	11/16				
Vascular invasion			**0.032**			0.187
Yes/No	21/64	13/16				
TNM,			**0.015**			0.284
I, IIA/ IIB, III or IV	39/46	11/16				
circ-ASH2L			**0.025**	1.741	1.075–2.821	**0.024**
High/Low	42/43	12/16				

# Forward LR method was applied during the multivariate cox regression analysis. The bold values indicate *P*-values less than 0.05

Furthermore, we found that PDAC patients with miR-34a low expression have significantly low overall survival rates compared with the counterparts (*P* = 0.0082) (Fig. 6e), and our result is consistent with other study in PDAC [29]; interestingly, Fig. 6f showed patients with combined high circ-ASH2L and low miR-34a expressions have the lowest survival rates compared to the only expression of circ-ASH2L or miR-34a (*P* = 0.0067).

Thus, we found that high circ-ASH2L expression is a risk factor for PDAC survival.

## Discussion

**Fig. 7:**
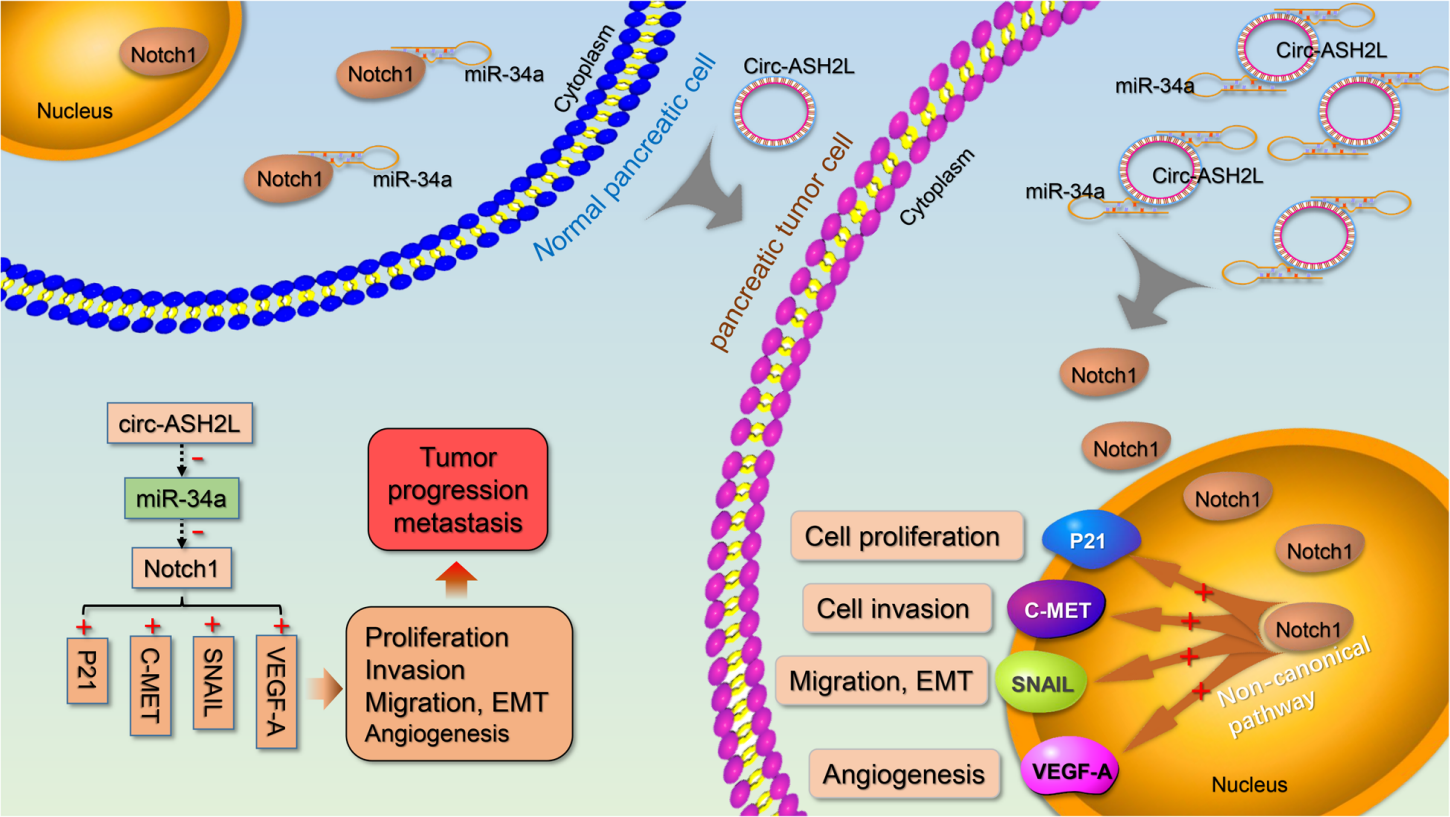
A schematic model of circ-ASH2L functions during tumor invasion

In this study, we screened out the circ-ASH2L, which was highly expressed in PDAC cells, based on our previous studies. Further research revealed that circ-ASH2L sponged miR-34a expression to promote Notch 1, thus promoting PDAC cell invasion, proliferation and angiogenesis. The roles of circ-ASH2L in PDAC were summarized in Fig. 7 based on the study. Furthermore, we found that high circ-ASH2L expression was corrected with poor prognosis of PDAC patients.

CircRNAs are a type of covalent closed circular noncoding RNAs that function similarly to other classic noncoding RNAs [30]. CircRNAs mainly regulate genes that modulate tumor growth, metastasis, proliferation, and chemoresistance through the circRNA–miRNA–mRNA axis in different kinds of tumors. Currently, although intense investigations of the roles of circRNAs in tumor progression have been performed, only a few studies have focused on circRNAs in pancreatic cancer. For example, circ_0006988 was found significantly elevated in pancreatic cancer tissues and plasma, its ectopic expression was related to PDAC progression and chemoresistance [31]. Therefore, exploring new diagnostic or prognostic markers in PDAC is important, and we believe that circ-RNAs are ideal tumor markers for PDAC. For decades, PDAC has been the most malignant among all tumors, with a 5-year overall survival rate less than 6%. Surgical resection is the main treatment for PDAC thus far. However, almost 80% of patients have lost the chance for a curative operation when they are admitted to the hospital for symptoms such as obstructive jaundice [32]. Thus, early detection or diagnosis is the most effective solution for PDAC treatment. Recently, an increasing number of circ-RNAs have been discovered and found to play important diagnostic or prognostic roles in different tumors. For example, aberrant expression levels of different circ-RNAs are found in ovarian cancer [33], lung cancer [34], renal cancer [35], gastric cancer and so on [36]. Pancreatic cells are rich in different enzymes, including different RNA enzymes, which results in extremely difficult RNA detection in frozen PDAC samples. In fact, in our previous studies, we found that most PDAC samples stored at − 80 °C were not suitable for RNA extraction because of RNA degradation unless the sample was placed into liquid nitrogen as soon as possible. Therefore, detecting tumor-related mRNAs, miRNAs or lncRNAs is difficult in frozen PDAC samples, which limits further application in clinical practice. However, circ-RNAs are stable in tumor tissues and can resist RNase degradation [37]. In this study, RNase R or Actinomycin D was used to treat pancreatic cells, and we found that circ-RNA expression was barely changed, but the expression of other RNAs, such as mRNAs, was reduced sharply. Therefore, we believe that an increasing number of tumor-related circ-RNAs may have important roles in PDAC research.

MiR-34a, a classic tumor suppressor, plays an important role in the initiation, progression and even therapy of many tumors [20]. MiR-34a was found to regulate many key genes that control the cell cycle, cellular proliferation, apoptosis, DNA repair and angiogenesis [19]. Ectopic miR-34a expression is believed to be a prognostic cancer signature. Based on previous studies, we found that miR-34a could active Notch 1 pathway, thus influence many classic genes like P21, c-Met, SNAIL and VEGFA, that may explain why circ-ASH2L promote the malignancy of PDAC. In addition, miR-34a was generally found to be positively regulated by various transcription factors, including p53, Tap73 or EST-like protein 1, of which the strongest inducer is p53 [20]. MiR-34a could also be negatively regulated by another transcription factor, p63 [38]. In this study, we revealed that miR-34a could also be negatively regulated by circ-ASH2L as a miRNA sponge. First, we found that the cellular functions of circ-ASH2L and miR-34a are nearly the opposite. By conducting gain-of-function or loss-of-function circ-ASH2L and miR-34a experiments, we found that circ-ASH2L promotes tumor progression via miR-34a. Furthermore, to detect the detailed regulatory mechanisms between circ-ASH2L and miR-34a, we performed bioinformatic analysis and discovered that each circ-ASH2L bound to two strong complementary sites in the seed region of miR-34a. Using qRT-PCR and dual-luciferase reporter gene assays, we confirmed that circ-ASH2L reduced miR-34a expression by acting as a ceRNA. Thus, our study identified a new mechanism of miR-34a regulation, which was not reported in other studies.

Our results suggest that ectopic circ-ASH2L may be an important marker in PDAC. Our in vivo assay showed that circ-ASH2L promoted tumorigenesis and tumor progression, and further correlation analysis in clinical PDAC samples showed that high circ-ASH2L was associated with lymphatic invasion or TNM stage. Further univariate and multivariate analyses suggested that high circ-ASH2L expression is an independent risk factor for PDAC survival. Treatment with miR-34a has already entered a clinical trial in liver cancer, and ectopic circ-ASH2L may also be an important factor in the diagnosis and prognosis of PDAC.

## Conclusions

We identified circ-ASH2L based on our previous studies and found that circ-ASH2L was abundant, stable and highly expressed in PDAC cells. By serving as a ceRNA for miR-34a, circ-ASH2L promoted tumor invasion, proliferation and angiogenesis by regulating miR-34a expression to active Notch 1 pathway. In the end, we found that high circ-ASH2L expression is related to tumor progression and is an independent risk factor for PDAC patient survival. Therefore, circ-ASH2L may be a useful biomarker in PDAC.

## Data Availability

Not applicable.

## Abbreviations

PDAC: Pancreatic ductal adenocarcinoma circRNAs Circular RNAs miRNAs microRNAs IP RNA-binding protein immunoprecipitation FISH Fluorescence in situ hybridization
