# Correction to: Circ-ASH2L promotes tumor progression by sponging miR-34a to regulate Notch1 in pancreatic ductal adenocarcinoma

**DOI:** 10.1186/s13046-021-01902-0

**Published:** 2021-04-16

**Authors:** Yan Chen, Zhonghu Li, Mengyun Zhang, Bo Wang, Jiaxin Ye, Yang Zhang, Di Tang, Dandan Ma, Weidong Jin, Xiaowu Li, Shuguang Wang

**Affiliations:** 1 Hepatobiliary Surgery Institute, Southwest Hospital, Army Medical University, Chongqing, China; 2 Hepatobiliary Surgery Department, 958 Hospital of PLA, Chongqing, Chongqing, China; 3 Dept. general surgery, Central Theater Command General Hospital of PLA, Hubei, China; 4 Department Rheumatology of Integrated Traditional Chinese and Western Medicine, Central Theater Command general hospital of PLA, Hubei, China; 5 Hepatobiliary Surgery & Carson International Cancer Shenzhen University General Hospital & Shenzhen University Clinical Medical Academy Center Shenzhen University, Shenzhen, China

**Correction to: J Exp Clin Cancer Res 38, 466 (2019)**

**https://doi.org/10.1186/s13046-019-1436-0**

Following publication of the original article [1], the authors identified some minor errors in image-typesetting in Fig. 4; specifically in Fig. 4a and Fig. 4h.

The corrected figure is given below. The correction does not have any effect on the results or conclusions of the paper.

**Fig. 4:**
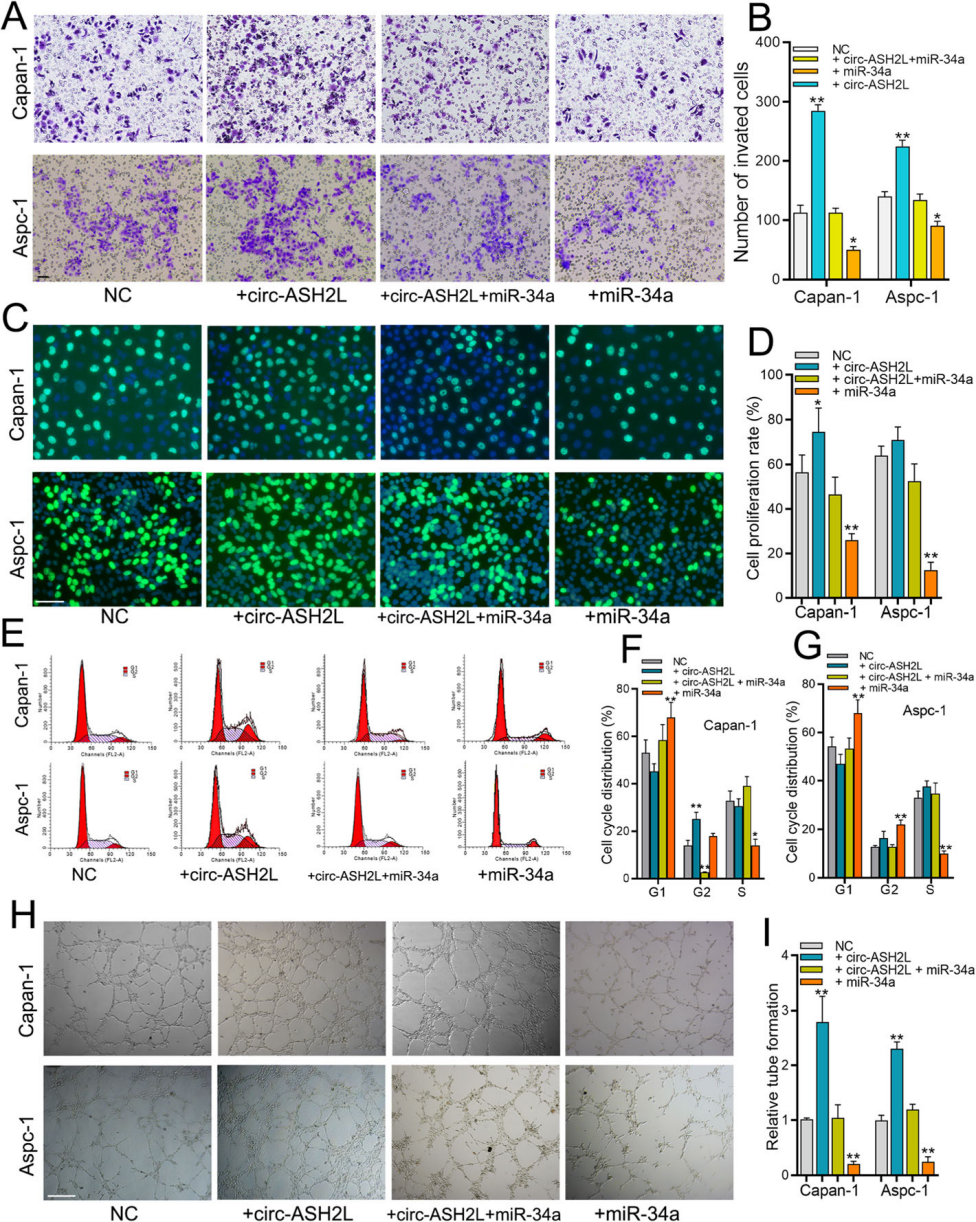
**a-b** The invasion abilities of indicate treated Capan-1 (**a**) and Aspc-1 cells (**b**) were measured by transwell assays. Scale bars = 50 μm. **c-d** The proliferation abilities of indicate treated Capan-1 (**c**) and Aspc-1 cells (**d**) were measured by EdU assays. Scale bars = 50 μm. **e-g** The indicate treated Aspc-1 and Capan-1 cells (**e**) were stained by propidium iodide and analyzed using flow cytometry, the statistical results of Aspc-1 (**f**) and Capan-1 (**g**) cells were showed in right column. **h-i** The in vitro angiogenesis abilities of indicated treated Capan-1 and Aspc-1 cells were measured by tube-formation assays of HUVECS cells (**h**), and the statistical result was showed in right column (**i**)
